# Automated Classification of Atherosclerotic Radiomics Features in Coronary Computed Tomography Angiography (CCTA)

**DOI:** 10.3390/diagnostics12071660

**Published:** 2022-07-08

**Authors:** Mardhiyati Mohd Yunus, Ahmad Khairuddin Mohamed Yusof, Muhd Zaidi Ab Rahman, Xue Jing Koh, Akmal Sabarudin, Puteri N. E. Nohuddin, Kwan Hoong Ng, Mohd Mustafa Awang Kechik, Muhammad Khalis Abdul Karim

**Affiliations:** 1 Programme of Diagnostic Imaging and Radiotherapy, Faculty of Health Sciences, Universiti Kebangsaan Malaysia (UKM), Kuala Lumpur 56000, Malaysia; mardhiyati@unisel.edu.my (M.M.Y.); a169010@siswa.ukm.edu.my (X.J.K.); akmal.sabarudin@ukm.edu.my (A.S.); 2 Programme of Medical Imaging, Faculty of Health Sciences, Universiti Selangor (UNISEL), Shah Alam 40000, Malaysia; 3 Imaging Centre, Institut Jantung Negara (IJN), Kuala Lumpur 50400, Malaysia; drahmadk@ijn.com.my (A.K.M.Y.); muhdzaidi@ijn.com.my (M.Z.A.R.); 4 Institute of IR4.0, Universiti Kebangsaan Malaysia (UKM), Bangi 43600, Malaysia; puteri.ivi@ukm.edu.my; 5 Faculty of Business, Higher College of Technology, Sharjah, United Arab Emirates; 6 Department of Biomedical Imaging, Faculty of Medicine, Universiti Malaya, Kuala Lumpur 50603, Malaysia; ngkh@ummc.edu.my; 7 Faculty of Medicine and Health Sciences, UCSI University, Persiaran Springhill, Port Dickson 71010, Malaysia; 8 Department of Physics, Faculty of Science, Universiti Putra Malaysia (UPM), Seri Kembangan 43400, Malaysia; mmak@upm.edu.my

**Keywords:** atherosclerotic plaques, CCTA, radiomic features, AutoML, TPOT, supervised

## Abstract

Radiomics is the process of extracting useful quantitative features of high-dimensional data that allows for automated disease classification, including atherosclerotic disease. Hence, this study aimed to quantify and extract the radiomic features from Coronary Computed Tomography Angiography (CCTA) images and to evaluate the performance of automated machine learning (AutoML) model in classifying the atherosclerotic plaques. In total, 202 patients who underwent CCTA examination at Institut Jantung Negara (IJN) between September 2020 and May 2021 were selected as they met the inclusion criteria. Three primary coronary arteries were segmented on axial sectional images, yielding a total of 606 volume of interest (VOI). Subsequently, the first order, second order, and shape order of radiomic characteristics were extracted for each VOI. Model 1, Model 2, Model 3, and Model 4 were constructed using AutoML-based Tree-Pipeline Optimization Tools (TPOT). The heatmap confusion matrix, recall (sensitivity), precision (PPV), F1 score, accuracy, receiver operating characteristic (ROC), and area under the curve (AUC) were analysed. Notably, Model 1 with the first-order features showed superior performance in classifying the normal coronary arteries (F1 score: 0.88; Inverse F1 score: 0.94), as well as in classifying the calcified (F1 score: 0.78; Inverse F1 score: 0.91) and mixed plaques (F1 score: 0.76; Inverse F1 score: 0.86). Moreover, Model 2 consisting of second-order features was proved useful, specifically in classifying the non-calcified plaques (F1 score: 0.63; Inverse F1 score: 0.92) which are a key point for prediction of cardiac events. Nevertheless, Model 3 comprising the shape-based features did not contribute to the classification of atherosclerotic plaques. Overall, TPOT shown promising capabilities in terms of finding the best pipeline and tailoring the model using CCTA-based radiomic datasets.

## 1. Introduction

According to the World Health Organization (WHO), cardiovascular diseases (CVDs) are the major cause of death worldwide, with an anticipated 17.9 million people dying from CVDs in 2019 [1]. The inflammatory disease atherosclerosis is defined as a localised accumulation of lipids, cholesterol, and other substances within the arterial wall. The plaque that forms inside the coronary artery restricts blood flow to the heart, increasing the risk of angina, myocardial infarction, and even death. Hence, atherosclerosis can be considered as a significant cause of CVDs or better recognized as a “silent killer” due to the difficulty in determining its occurrence, especially among asymptomatic patients. Generally, atherosclerotic plaques consist of the calcified, mixed, and non-calcified types of plaques in which all have their specific characteristics. However, the presence of non-calcified atherosclerotic plaques inside the coronary arteries is strongly linked to an elevated risk of a cardiovascular event [2]. This is because non-calcified atherosclerotic plaques are more prone to rupture, which may result in thrombosis [3]. Therefore, it is important to have timely, cost-effective, and accurate diagnoses.

Prior research [4,5,6] have used coronary computed tomography angiography (CCTA) to focus on the traits and morphology of high-risk atherosclerotic plaques connected to acute coronary syndrome (ACS), such as positive remodelling, low attenuation, patchy calcification, and the napkin-ring sign. Along with CCTA, other imaging modalities such as positron emission tomography (PET), magnetic resonance coronary angiography (MRCA), intravascular ultrasound (IVUS), optical coherence tomography (OCT), and others are useful for evaluating coronary atherosclerotic plaques [7,8,9]. Notably, CCTA has irreplaceable advantages over other examinations. Notably, CCTA provides unbeatable benefits over other tests. First off, CCTA can swiftly offer insightful diagnostic data. Second, CCTA is a very non-invasive procedure with modest practitioner needs and a high degree of clinical applicability when compared to invasive coronary angiography (ICA), IVUS, and OCT. In comparison to MRCA and PET, CCTA also provides a higher spatial resolution for identifying the characteristics of high-risk plaques, more stable image quality [10] and cost-effective imaging technology that allows a clinician to quickly analyse plaque burden, especially in low- and intermediate-risk patients [11,12]. In addition, research comparing the diagnostic efficacy of CCTA to ICA indicated that CCTA had higher sensitivity, specificity, positive predictive value (PPV), and negative predictive value (NPV) for diagnosing coronary artery stenosis [13,14].

The current CCTA examination, on the other hand, has the disadvantage of relying only on the qualitative assessment of cardiologists as well as radiologists. As a result, existing subjective variability may have an impact on diagnosis outcomes and the final treatment plan decision [15]. This is because some important diagnostic information may be overlooked since the visual detection of the plaque characteristics is highly dependent on the expertise level of a cardiologist or radiologist. Moreover, the process of interpreting CCTA images manually by a radiology expert is also considered tedious and time-consuming [16]. Therefore, it triggers researchers worldwide to explore the automated machine learning approaches in the radiomics studies to automate the process of classifying the atherosclerotic plaques within the coronary arteries as either normal, calcified, non-calcified, or mixed plaques. In recent years, the emergence of high-throughput computing and the availability of big data has enabled researchers to explore the power of radiomics as well as machine learning (ML) in the field of medical imaging. It is an analytic approach based on computer algorithms.

Radiomics, on the other hand, is the technique of obtaining multiple quantitative features of digital medical images by converting it into high-dimensional data [17,18,19,20]. Radiomics is a combination of the term ‘radio’ which indicates medical images, and the term ‘omics’ which refers to various fields including genomics and proteomics that contribute to our understanding of various medical conditions [21]. The relationship between radiomics and machine learning is bilateral. It is inevitably true that machine learning applications need quantitative radiomic features to train and build the models, whereas the field of radiomics requires machine learning tools for intelligent data analysis [22]. Previous research conducted has used random forest algorithm to construct conventional and radiomics models, respectively, to forecast the stenosis plaques using CT-FFR information [23]. The study showed improved performance of the radiomics model in identifying the ischemic coronary stenosis plaques compared with the conventional model. The detection of both obstructive (50% stenosis) and non-obstructive (50% stenosis) lesions with an AUC of 0.94 was demonstrated in 2015 by Kang et al. [24] via machine learning. In addition, Freiman et al. [25] observed an AUC that varied from 0.78 to 0.94 for detection of mild stenosis 30% to severe stenosis 70% using a deep sparse autoencoder—mixed structure regularization technique in 90 patients.

More recently, in the multi-center CT Evaluation by Artificial Intelligence for Atherosclerosis, Stenosis, and Vascular Morphology (CLARIFY) study, level 3 (L3) readers and AI were tested for their ability to detect coronary artery stenosis on CCTA [26]. The researcher study found a 99.7% accuracy rate for recognizing >70% stenosis and a 94.8 accuracy rate for detecting >50% stenosis. The mean maximal diameter stenosis difference between AI and L3 readers among the vessels under analysis was only 0.8 percent. The categorization of Coronary Artery Disease Reporting and Data System (CAD-RADS) in comparison to L3 readers was also investigated using AI analysis. AI scored CAD-RADS in accord with readers in 78% of scans and within 1 category in 98% of scans. Griffin et al. [27] subsequent’s investigation of a multi-center cohort of patients having core-lab quantitative invasive angiography (QCA) revealed that AI CCTA had higher diagnostic accuracies than QCA for identifying >50 percent stenosis (AUC 0.88) and >70 percent stenosis (AUC 0.92) than QCA.

### Auto Machine Learning (AutoML)

Machine learning however is often met with challenges such as the performance of the machine learning approaches and is susceptible to various decision parameters, including the selection of algorithms, training procedures, and hyperparameters of all components. Usually, users need to self-select manually and optimize numerous parameters of each machine learning algorithm. They frequently have to go through a series of trials and errors to find the best neural architectures and parameters for a given dataset. Jordan et al. in 2015, also addressed the issue of the challenges of hyperparameters tuning in maximizing the performance of the machine learning model [28].

Automated machine learning (AutoML) is a tool that automates the process of developing a machine learning model that has lately gained popularity. AutoML libraries come in a variety of environment including Auto-WEKA [29], Tree-based Pipeline Optimisation Tool (TPOT) [30,31], Auto-Sklearn, and others [32,33,34]. In this context, AutoML runs through a dataset and suggests the most optimum algorithm with the parameters set. The use of AutoML, for example, improves the performance of prediction outcomes while removing the requirement for researchers to go through a series of trials and errors to find the best method for a given dataset. These challenges are considered as an opportunity to integrate the AutoML concept into the field of medical imaging.

AutoML is the process of automating the application of machine learning to real-world situations from start to the end. AutoML can be explained mathematically as follows [31]:

COsOP+2N.G(f1,f2)PNM+∑m′∈M∑r∈RP(〈m′.r|m〉P(r+y.v(m′)))

Here

${}^{O_{P}}$ is the default pre-defined operation set;
${}^{O_{s}}$ indicates the operations selected by the algorithms;
$G\left(f_{1},f_{2}\right)$ represents the generator function for developing new features;
*N* is the number of features selected;
*N_M_* = maximum number of features to be chosen.

Data pre-processing automation is defined as a series of activities chosen ${}^{O_{s}}$ from a default ${}^{O_{P}}$ operational set and performed on a data set. The features are extracted by calculating and producing new $G\left(f_{1},f_{2}\right)$ dependent pairs using the appropriate features ($2_{N}$) from the data source. The model selection and hyperparameter optimization work to identify the ideal parameter configuration from an endless search region or learn from prior models developed for specific objectives. The stochastic learning approach that has been utilised to limit the configuration space for numerous years is represented by the last term of the equation [35].

Certainly, this AutoML concept is still considered new in the field of medical imaging. However, in the field of brain age prediction, Dafflon et al. in 2020 have proven that TPOT as an AutoML library could be used to find a suitable machine learning model that had significantly better performance than a standard brain age prediction model, Relevance Vector Regression (RVR) [36]. Apart from that, TPOT demonstrated the promising predictive result even though given by large feature spaces and mixed data types consisting of demographic, clinical, and biomarker data [37].

**Figure 1:**
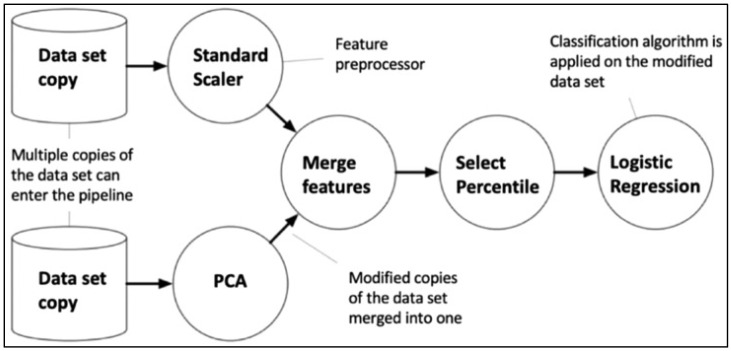
Tree-based pipeline from TPOT. Reprinted with permission from Ref. [37]. 2019, Oxford University Press.

The whole TPOT configuration comprised 11 classifiers, 14 feature converters, and 5 feature selectors, all of which worked together with TPOT to create the best pipeline out of all of these combinations. The TPOT pipeline often begins with one or more copies of the full data set at the top of the tree structure, then moves on to function transformations or feature selectors, as shown, or the ML method. The operators then make adjustments to the original data set before passing it on to the next operator in the tree. Figure 1 depicts a TPOT sample tree-based pipeline. Each circle represents a machine learning operator, with the arrows indicating the data flow direction.

From such existing development of TPOT in the medical field as mentioned, it is possible to adopt TPOT in developing a machine learning model that can classify the type of atherosclerotic plaques.

TPOT is one of the examples of automated machine learning which have been developed and emerged to automatically optimize parts of the machine learning pipelines [38]. This method is currently accessible to all healthcare professionals, especially non-experts’ users since it is more user-friendly than conventional machine learning as it already automates the process starting from running the raw input dataset until the development of a machine learning model including the optimization process. Furthermore, AutoML analyses a dataset and recommends the best method based on the hyperparameters [29]. As a result, it eliminates the requirement for researchers to conduct a series of trials and errors to determine the optimum method for a given dataset. As far as we study from the previous literature, there we limited research done on the application of automated machine learning especially using TPOT algorithm in developing automated classification of atherosclerosis model using CCTA radiomics features. This research also was conducted using real patient database covering all CADRADS categories of atherosclerosis in one of the main cardiac centre of Malaysia. Therefore, in this study, our aim was to extract the radiomic features of the atherosclerotic plaques from CCTA images and evaluate the performance of automated machine learning model using TPOT algorithm in classifying the atherosclerotic plaques. This research contribute more into the radiomics features quantification of atherosclerosis using semi-automated segmentation in soft tissue windowing viewing setting of CCTA images. The research methodology, results, discussion, and conclusion on the diagnostic performance of each type of radiomic features quantification in the classification of the atherosclerotic plaques was evaluated.

## 2. Materials and Method

As per evaluation, all data including CCTA images, clinical information related to subjects and cardiologist as well as radiologist report were collected from the PACS system at the Imaging Department of National Heart Institute, Kuala Lumpur, Malaysia or also known as Institut Jantung Negara (IJN), Malaysia. In IJN, the cardiologist also involved in the radiology images reporting as well as the radiologist. An ethics approval (IJNREC/496/2021) has been obtained from the IJN Research Ethics Committee (IJNREC) on 14 April 2021 for this retrospective study.

### 2.1. Study Population

**Figure 2:**
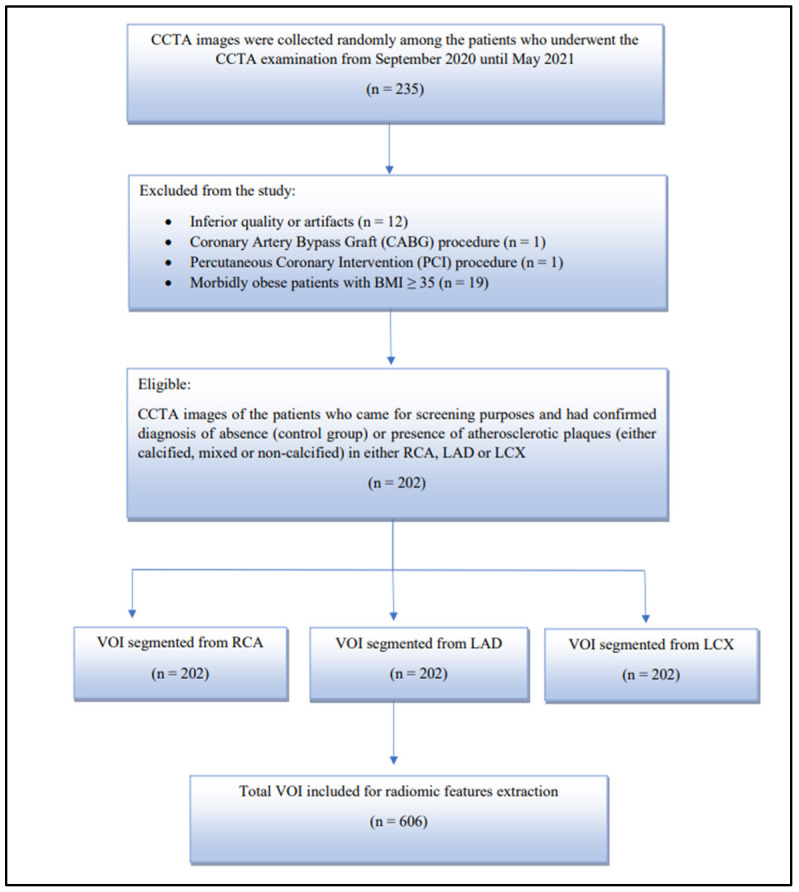
Overall flow of patient selection.

In total, 235 patients who underwent the CCTA examination from September 2020 until May 2021 were choose randomly in this study. In total, there were 202 subjects whose CCTA fit the inclusion and exclusion criteria. The main inclusion criteria in this study are those subjects who went for CCTA examination for screening purposes. The subjects were either normal (labelled as a control group) or had confirmed the presence of atherosclerotic plaques in either right coronary artery (RCA), left anterior descending artery (LAD), or left circumflex artery (LCX) in diagnosis. Only CCTA images with constant imaging parameters (kV range of 100–140 kV) were included in this study. The exclusion criteria including those with inferior image quality or present of artifacts, patients who had undergone coronary artery bypass graft (CABG), or percutaneous coronary intervention (PCI) procedure, and morbidly obese patients (BMI ≥ 35). The overall flow of patient selection is shown in Figure 2.

### 2.2. Workflow

**Figure 3:**
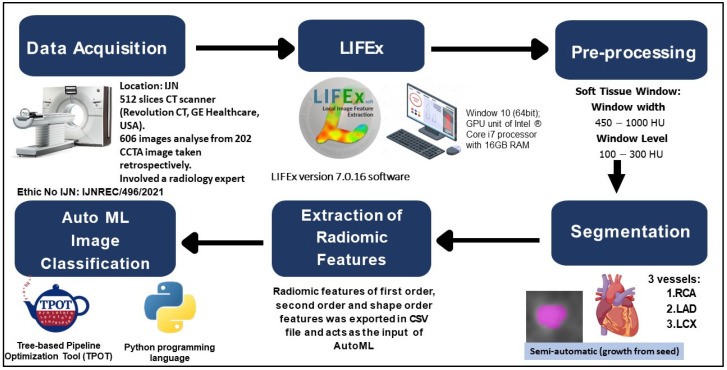
Overall research workflow.

Data acquisition was the first step in a radiomic investigation, which was followed by image pre-processing, segmentation, feature extraction and automated classification. Figure 3 depicted the stages of the process, starting with the image acquisition phase and ending with the use of TPOT to find the best classifier for the classification of atherosclerotic plaques.

On the axial view of CCTA multislice images, semi-auto segmentation of the coronary arteries was conducted in this study. A cardiologist from IJN with at least 10 years of expertise evaluating CCTA pictures and validate the segmentation process. From the segmented regions, radiomic features were extracted. Following that, the extracted radiomic features were fed into the TPOT to identify the best classifier. The TPOT recommended the best classifier to classify atherosclerotic plaques.

### 2.3. Image Acquisition

The CT machine used at IJN is a GE Medical System, Brand Revolution 512 United States of America (U.S.A.). CCTA images are stored on an external hard disk via offline software namely DICOM SIEMENS Syngo.Via Software, (Erlangen, Germany). Technically, the image produced is through a retrospective electrocardiogram (ECG) type imaging technique with a CT machine voltage rate of 120 kVp using the specified parameters. Smart mA (50–430) automatic exposure control, gantry rotation time of 280 ms with one gantry rotation with scan of the entire heart image scanned in one gantry rotation and one heartbeat. Volume scans were dynamically taken with a slice thickness of 0.63 mm with minimal overlap distance with noise index of 20. Volume scans were taken dynamically with a scan range between 120–160 cm starting at the bifurcation branch of the trachea until the diaphragm covers the entire heart with a heart scan size area value of 14 cm.

Throughout the scan, the ECG detection technique was set at 40–70% at the peak of the R-R interval (the interval between one heartbeat to another heartbeat). In addition, the pitch value of 0.25 mm/s is used because the scan mode used is spiral. For examination with several phases of the heart, the contrast agent used is 55–60 mL (5 mL/s) given by Care bolus with a pressure value of 2300 PSI when the image concentration reaches 100 Hounsfield Units (HU). Once the imaging process is completed, the image reconstruction process is retrospectively performed by obtaining images at 70 bpm values obtained around 70–80% RR phase and if heart rate exceeds 80 bpm the obtained phase around 30–40% RR period will be processed.

Furthermore, the Trak care 2014 Internet Explorer was used to examine and obtain the patients’ radiological reports. In addition, patient information (age, gender, ethnicity, weight, and height) was collected from the imaging request form, and BMI was calculated. Table 1 summarises the demographic characteristics in detail.

### 2.4. Pre-Processing

The CCTA image was loaded into LIFEx (version 7.0.16) software (Orsay, France) [39]. The image layout was adjusted for axial view images and was magnified up to 10 times zooming enlargement for better visualization. Furthermore, the window width (WW) and window level (WL) were adjusted to achieve soft-tissue window settings. The range of WW used was between 500 to 1000 HU whereas the range of WL was between 100 to 300 HU.

### 2.5. Segmentation Process

**Figure 4:**
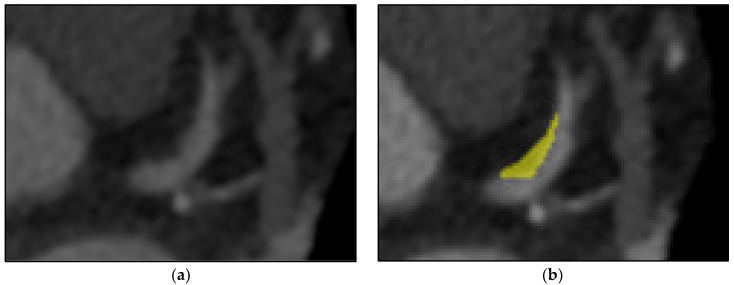
(**a**) Before segmentation of proximal LAD and (**b**) after segmentation of non-calcified lesion on proximal LAD using semi-automated (growth from seed) type of segmentation which was colored into yellow colour.

The LIFEx (7.0.16 version) programme was used to semi-automate the segmentation of all CCTA pictures. The RCA, LAD, and LCX are the three primary coronary arteries that were segmented. For each image, a 3D brush (a semi-automatic segmentation technique) was used to draw the volume of interest (VOI) on the spesific identified vessels. As a first step, the reader marks the area of interest. In the second step, semi-automated segmentation done on the next slice and repeat again to the next slice. Then, the LIFEx software will automatically tries to establish the next slice of segmentation area based on luminal intensity values by growth from seed approach. The segmentation area is subsequently presented to the reader for manual correction. Figure 4 showed the CCTA images of the pre-segmentation and the post-segmentation process on non-calcified lesions on proximal LAD.

**Figure 5:**
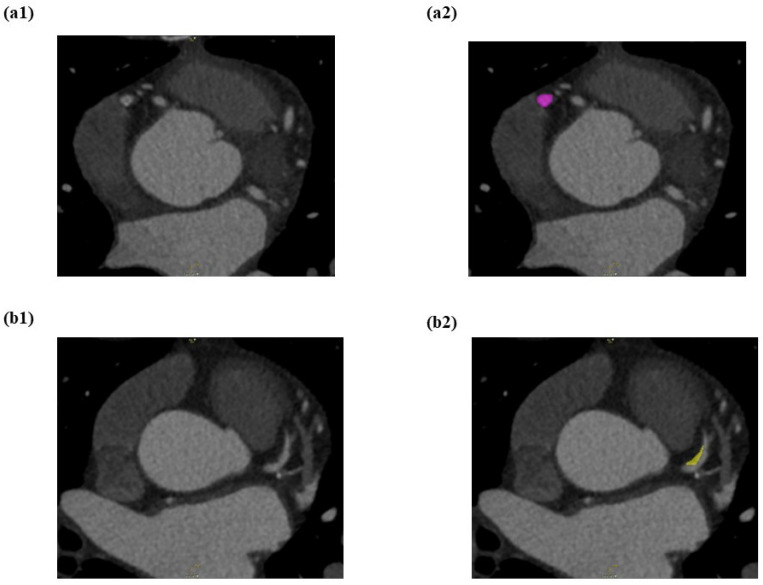

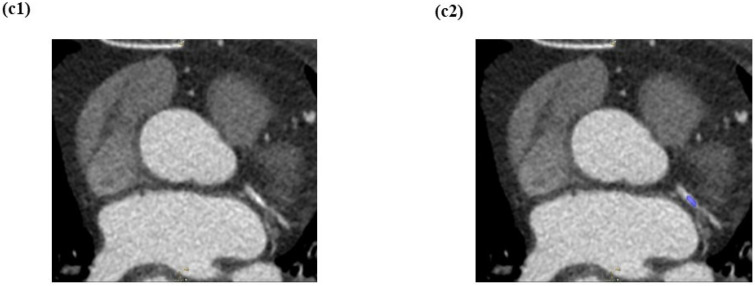
LIFEx software is used to perform semi-automated segmentation on RCA, LAD, and LCX. (**a1**) Mid RCA with a mixed calcified atherosclerotic plaque seen. (**a2**) The mixed calcified plaque was enclosed by the VOI placement (pink colour) on the mid RCA. (**b1**) Proximal LAD with a non-calcified atherosclerotic plaque was seen. (**b2**) The non-calcified plaque was surrounded by the VOI placement (yellow colour) on the proximal LAD. (**c1**) Proximal LCX with a calcified atherosclerotic plaque was observed. (**c2**) The calcified atherosclerotic plaque was surrounded by the VOI placement (blue colour) on the proximal LCX.

In this study, the proximal, mid, or distal parts of the RCA, LAD, and LCX where regardless the plaque is present or not is segmentized. The segmentation of the normal coronary artery was identified as a control group. Three adjacent slices of the area of interest were used to segment the VOIs, which were then stored in the system. The segmentation was verified by an independent observer, a senior cardiologist at IJN. A detailed explanation of the segmentation method was shown in Figure 5.

### 2.6. Feature Extraction Process

After each coronary artery had been segmented, the radiomic features were retrieved directly. A total of 29 first order features, 5 shape order features, and 31 second order features were retrieved from each VOI of the RCA, LAD, and LCX in this work. There were 13 conventional indices features and 16 discretized indices features in the first order features. Grey level co-occurrence matrix (GLCM) features, Grey level run matrix (GLRLM) features, neighbourhood grey-level difference matrix (NGLDM) features, and grey-level zone length matrix (GLZLM) features were among the second order features obtained. Based on the first order, second order, and shape order features, the extracted radiomic features were sorted and recorded in a CSV file format. The data were then manually cleaned to remove the Not a Number (NaN) entries. The TPOT employed the final ‘clean’ dataset for each order of radiomic characteristics as an input dataset. It was employed by TPOT to find a good classifier that could be automated. Table 2 summarises the radiomic characteristics derived in this investigation in detail.

### 2.7. Tree-Based Pipeline Optimisation Tool (TPOT) Multiclass Classification of Atherosclerotic Plaques

**Figure 6:**
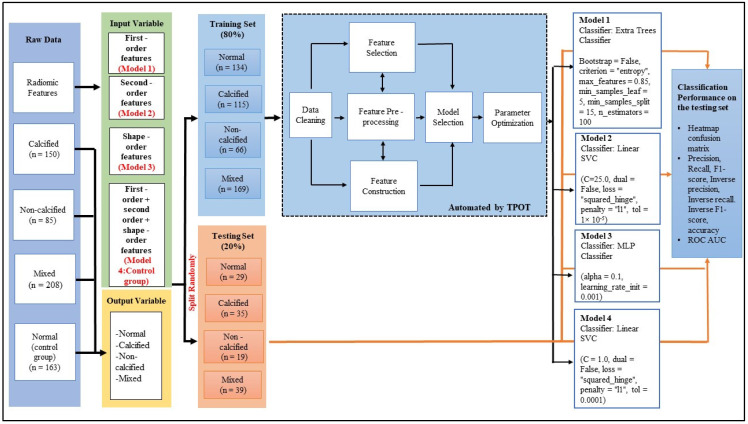
Pipeline search by TPOT. Initially, raw data was split into input and output variables.

TPOT version 0.11.7 [40] was an open-source library to perform AutoML in Python Package. For each TPOT-based model, the selection was performed with different input datasets which were named Model 1 for the first order features, Model 2 for the second order features, Model 3 for shape order features, and Model 4 as a combined group of first order, second order, and shape order features which acted as a control group. TPOT was used to import the data from the CSV file. Input and output variables were separated from the dataset. The radiomic characteristics were used as input, whereas the radiologist’s diagnostic of ‘calcified, non-calcified, mixed, or normal’ was used as output for this supervised form of machine learning technique. Following that, this dataset was randomly split into training (*n* = 485) and testing (*n* = 121) sets in an 80:20 ratio. This is supported by the study that showed an increased training data volume enhances the algorithm performance of classification [41]. If the training sets are too few, it may cause our models to become underfitting. TPOT used genetic programming on the training set to search through the machine learning classifiers through a streamlined process of data cleaning, feature selection, feature processing, feature creation, model selection, and hyperparameter optimization. The default settings for TPOT were used (generations = 100, population size = 100, cv = 5, verbosity = 2, *n* jobs = 1, random state = 0). Following that, Python code was used to generate information about the classifier provided by TPOT, as well as its parameter settings. Figure 6 depicted the TPOT pipeline’s workflow in further detail.

Four types of ML models (Model 1, 2, 3, and 4) with different input variables were constructed, respectively. Model 1 was constructed using the features under the first order features group. Model 2 was built using the features under the second order features group. Model 3 was based on shape order features to construct its classification model and finally, the Model 4 was a control model that used all the extracted features mentioned to build the classification model. The process of building the classification model was summarized as follows: (1) The classifier and its parameter were set based on the result obtained from the TPOT run. (2) The training dataset was used to train with the corresponding classifier and its parameters. (3) After completing the training process, the prediction pipeline on the classification of atherosclerotic plaques was made directly on the testing data for unbiased estimation of classification performance.

### 2.8. Statistical Analysis

Statistical analysis was performed using SPSS version 26.0 (SPSS Inc., Chicago, IL, USA) and Python (version 3.8.8) programming packages Sklearn, Pandas, and Numpy, respectively. Data including gender, ethnicity, the patients’ distribution based on CAD-RADS, and the dataset distribution based on the type of atherosclerotic plaques were expressed as frequency and percentages in qualitative assessment. Data including age, body mass index (BMI), total dose length product (DLP), heart rate, and the volume of contrast medium were expressed as (mean ± standard deviation) for quantitative assessment. The data of the patients’ demographic and image acquisition characteristics, as well as the patients’ distribution based on CAD-RADS, were tabulated in Table 1. Furthermore, all figures including the 4 × 4 heatmap confusion matrix, receiver operating characteristic (ROC) and area under the curve (AUC) were generated using Python Matplotlib and Seaborn programming packages. Then, this dataset was divided randomly into training and testing sets, respectively, in the proportion of 80:20. The process of data cleaning, feature selection, feature processing, feature construction, model selection, and parameter optimization was automated by TPOT and was performed on the training set, until a pipeline (classifier) optimized for the parameters of a particular model was selected. Lastly, the selected pipeline was applied and fitted on the testing set to evaluate the classification performance of a particular model. The results of precision (PPV), recall (Sensitivity), F1 score, inverse recall (Specificity), inverse precision (NPV), inverse F1 score, and accuracy were tabulated in Table 3 to evaluate the classification performance of each type of plaque in each classification model with different input variables.

## 3. Results

In this study, we employed CCTA images from IJN retrospectively and evaluate the applicability of AutoML, specifically TPOT in classifying the atherosclerotic plaques based radiomics features. All 202 CCTA images were successfully segmented which consisted of 68.3% males and 31.7% females with a mean age of 58.84 ± 9.497 years old from 3 ethnicities which were Malay (51.5%), Chinese (22.8%), Indian (25.2%), and others (0.5%) as shown in Table 1. The mean body mass index (BMI) was 26.81 ± 3.746 kg/m^2^. The morbidly obese patients with BMI ≥ 35 were excluded from this study. This is because photographs of morbidly obese people are more sensitive to the artefact, lowering image quality significantly [42,43]. Image quality is one of the key components that influence image segmentation [44]. Among them, 6 (3.0%) were grouped as CAD-RADS 0, 9 (4.5%) were categorized as CAD-RADS 1, 54 (26.7%) belonged to CAD-RADS 2, 36 (17.8%) were reported as CAD-RADS 3, 95 (47.0%) were categorized as CAD-RADS 4, and another 2 (1.0%) were reported as CAD-RADS 5. For the CCTA image acquisition characteristic, the total dose length product (DLP) was 322.00 ± 167.926 mGycm, the patients’ heart rate was 70.20 ± 10.522 bpm, and the volume of contrast medium used was 59.57 ± 2.347 mL.

Segmentation is crucial to maintain the reproducibility for CCTA images-based radiomics analysis. A semi-auto segmentation approach was preferable to be used in this study since our dataset was large and the manual one is not preferable to be used as it is time-consuming. This is supported by a study that found out that manual segmentation is prone to interobserver variability and is considered tedious especially if the dataset sample is large [45]. Furthermore, the accuracy of the fully automated segmentation cannot be guaranteed although it improves a lot in terms of efficiency.

In this study, 606 VOI were segmented from the CCTA images. Among them, 163 (27%) were within the normal group, 150 (25%) were grouped as calcified plaque, 85 (14%) were categorized as non-calcified plaque, and another 208 (34%) were grouped as mixed plaque. The labelling of the VOI as either ‘calcified’, ‘non-calcified’, ‘mixed’ or ‘normal’ as reported in the radiologist report was important in this study because they acted as the output variable for this supervised type of machine learning technique.

Our study explored the use of TPOT, an AutoML tool to search for the most optimum pipeline in building the radiomic-based ML models. This is because TPOT automates the process of feature selection, feature pre-processing, feature construction, model selection, and parameter optimization [37]. Moreover, based on the study by Tan et al. in 2020, the application of TPOT can prevent the uncertainty of selecting the machine learning classifiers manually [46].

**Figure 7:**
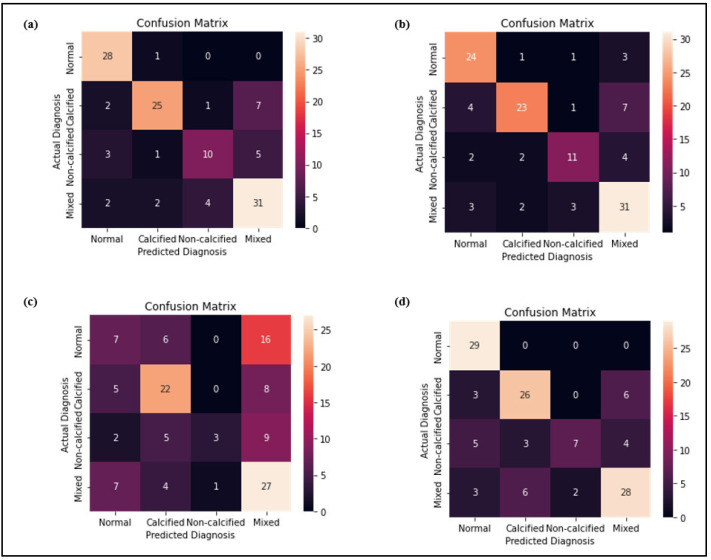
Heatmap confusion matrix for (**a**) Model 1, (**b**) Model 2, (**c**) Model 3 and (**d**) Model 4. Each column of the matrix represents the occurrence in a predicted class, whereas each row represents the occurrence in an actual class.

Based on Figure 7, there were four types of classification models (Model 1, 2, 3, and 4) built using different types of input variables but with a similar type of output variable. With the default TPOT settings (100 generations with 100 population size) applied in this study, TPOT has evaluated 10,000 pipeline configurations before selecting the most optimum pipeline. In this study, it is shown that TPOT suggested different classifiers for each model that has the dataset consisting of a different order of radiomic features as the input variable. In this study, it is shown that TPOT suggested different classifiers for each model that has the dataset consisting of a different order of radiomic features as the input variable. This is best evidenced as the classifier suggested by TPOT are Extra Trees Classifier for Model 1, Linear SVC classifier for Models 2 and 4, and lastly Multi-layer Perceptron (MLP) classifier for Model 3 after screening all four types of input data matrix by the TPOT pipeline process. It is noted that although the suggested classifier was similar in both Models 2 and 4, the optimization parameters were different among both. Overall, this represents that TPOT has personalized the best model for each data array.

Figure 7 shows the 4 × 4 heatmap confusion matrix for each model. Each column of the matrix represents the occurrence in a predicted class, whereas each row represents the occurrence in an actual class. The total correct values predicted per class or better known as true positive (TP) value was represented by the diagonal elements in the confusion matrix. The highest number was represented by the lightest color as shown in the color bar on the side. Based on the analysis of the confusion matrix, Model 1 showed the lightest color of diagonal element and the highest TP value (N:31) in classifying mixed plaque and almost nearly good performance as Model 4 in classifying the normal and calcified plaques. The confusion matrix of Model 2 showed the highest TP value (N:11) which was represented by the lightest color of the diagonal element specifically in classifying non- calcified plaques as compared to other models. In contrast, Model 3 had demonstrated the darkest color of diagonal elements, and the lowest TP values with (N:7) for normal, (N:22) for calcified, (N:3) for non-calcified, and (N:27) for mixed plaques.

The performance metrics including recall (Sensitivity), precision (PPV), F1 score, inverse recall (Specificity), inverse precision (NPV), inverse F1 score, and accuracy were used to have a better understanding of the models’ classification performance in classifying each type of plaques. The multi-class classification as shown in this study was broken down into a series of binary problems for each class (normal versus the rest, calcified versus the rest, non-calcified versus the rest, and mixed versus the rest) using One-vs-Rest or also called as One-vs-All approach in the machine learning field. For instance, in terms of classifying the calcified plaques, the data from the class ‘calcified’ were treated as ‘positive’, whereas the data from other classes were treated as ‘negative’, and the same concept was applied for the classification of other types of plaques too. This is because researchers [47] have found that most of the classification metrics were defined and applicable for binary class classification only.

When classifying the atherosclerotic plaques, we are mainly interested in the classification model’s ability to classify both positive and negative cases for each class. This is because we want to minimize the mistakes of missed and falsely classifying each class of plaques. Recall, precision, and F1 score were important performance metrics used to evaluate the performance of classifying the positive class correctly from the rest. Recall also referred to sensitivity, characterized the model’s ability to predict the positive correctly among the whole proportion of real positive cases. It was often accompanied by the precision, also called PPV metrics, that denotes the proportion of predicted positive cases that were correctly real positives. However, there was often a trade-off between recall and precision. Hence, the balance between precision and recall was best observed using the F1 score, which was the harmonic mean between precision and recall. The range of the F1 score was between 0 and 1, with 0 being the worst balance between precision and recall, and 1 being the vice versa. Apart from that, inverse recall, inverse precision, and inverse F1 score were important performance metrics used to evaluate negative case classification performance. Inverse recall measures the proportion of true negatives (TN) out of all actual negative cases, whereas inverse precision measures the proportion of TN out of all the cases classified as negative. The inverse F1 score is the harmonic mean between inverse recall and inverse precision. Hence, it is a supplement to the F1 score, which is commonly used in machine learning to assess positive case classification performance.

Table 3 has summarized the diagnostic performance of each model in classifying each type of plaque using the performance metrics as discussed. In terms of classifying the normal arteries from the abnormal ones, Model 1 with the highest accuracy of 0.92 achieved the best balance between recall (0.97) and precision (0.80) which resulted in the highest F1 score (0.88), as well as the best balance obtained between inverse recall (0.90) and inverse precision (0.99) which resulted in the highest inverse F1 score (0.94) among other models. A similar trend is observed in the classification of the calcified plaque where Model 1 with the highest accuracy of 0.87 showed the best balance between recall (0.71) and precision (0.86), as well as the best balance between inverse recall (0.95) and inverse precision (0.87) which resulted in the highest F1 score (0.78) and the best inverse F1 score (0.91), respectively. Similarly, in terms of classifying the mixed plaque, Model 1 with the highest accuracy of 0.82 demonstrated again the highest F1 score (0.76) and inverse F1 score (0.86), respectively, because of the best balancing achieved between recall (0.79) and precision (0.72), as well as the best balance between inverse recall (0.84) and inverse precision (0.89) compared to the others. In terms of classifying the non-calcified plaque, the highest F1 score of 0.63 was reported in Model 2 because of the best balance achieved between recall (0.58) and precision (0.69) as compared to others. However, Models 1, 2, and 4 were reported to have similarity in terms of the highest accuracy of 0.87 as well as the highest inverse F1 score of 0.92 because of the best balance achieved between inverse recall (Model 1: 0.94; Model 2: 0.94; Model 4: 0.98) and inverse precision (Model 1: 0.94; Model 2: 0.94; Model 4: 0.98), respectively. Therefore, we concluded that Model 2 comprising the second-order features had the best performance in the classification of non-calcified plaques which were revealed clearly from the overall results of the F1 score, inverse F1 score, and accuracy.

Conversely, Model 3 with the lowest accuracy of 0.62 showed the lowest F1 score (0.28) because of the balance achieved between both the lowest recall (0.24) and precision (0.33) values, and also the lowest inverse F1 score (0.74) obtained due to the balancing between the lowest inverse recall (0.79) and inverse precision (0.70) in classifying the normal arteries from the abnormal ones. A similar worst performance was observed in classifying the calcified plaques in which Model 3 with the accuracy of 0.68 had the lowest recall (0.63) and precision (0.59) that caused the lowest F1 score (0.61). Model 3 was also reported to have the lowest inverse recall (0.71) and inverse precision (0.74) that caused the lowest inverse F1 score (0.72) in classifying the calcified plaques. In terms of the classification of non-calcified plaque, although Model 3 with the lowest accuracy of 0.78 showed a slightly higher precision (0.75), its F1 score of 0.26 was still the lowest because of the lowest recall of 0.16 compared to other models. Similarly, the lowest inverse F1 score of 0.87 was reported in Model 3 in classifying the non-calcified plaque, because of the balance achieved between inverse recall (0.98) and inverse precision (0.78). In terms of classifying the mixed plaque, Model 3 with the accuracy of 0.57 reported the lowest F1 score (0.55) and inverse F1 score (0.59), respectively, because of the balance achieved between both the lowest recall (0.69) and precision (0.45), as well as the balance between both the lowest inverse recall (0.49) and inverse precision (0.73) compared to other models. To sum it up, it can be suggested that Model 3 which represents the shape-order features not be used as the input for developing the atherosclerotic CCTA image classification model. This is because the shape features are not considered much during the segmentation process; however, we only focus on extracting the radiomics features from 3 subsequent slices of an axial view of CCTA images to extract the information from the images.

**Figure 8:**
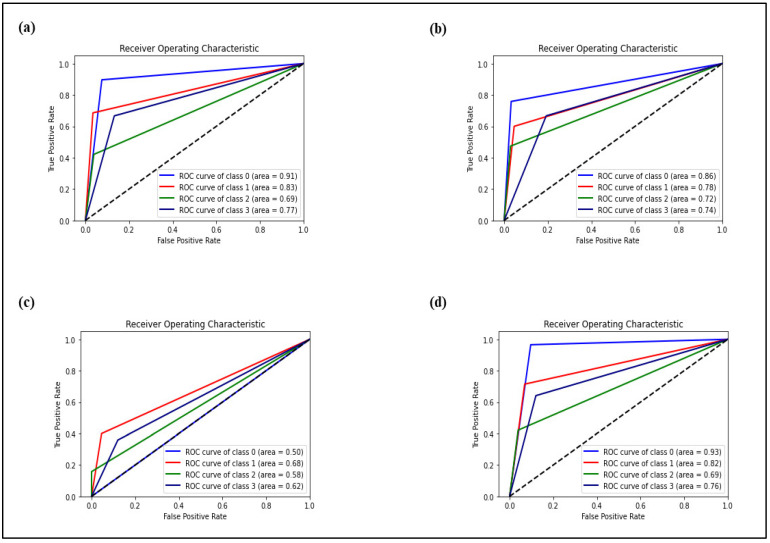
ROC curve for (**a**) Model 1, (**b**) Model 2, (**c**) Model 3 and (**d**) Model 4.

Figure 8 has shown the receiver operating characteristic (ROC) area under the curve (AUC) of each model in classifying each type of plaques versus the rest. The ROC curve was used to report the trade-off between true positive rate (TPR) and false-positive rate (FPR). Generally, the closer the curve is to the top left side as shown in the ROC curve, the higher the sensitivity and the lower the FPR of the classifier. AUC was used to determine the discriminating power of the ROC curve. The range of the AUC value was between 0 and 1, with the higher the value is, the better the predictive accuracy. The superior performance of Model 1 which represents the first-order features was shown with the highest AUC of 0.83 and 0.77 in classifying the calcified and mixed plaques, respectively. In terms of classifying the normal arteries, Model 4 as a control group showed the highest AUC of 0.93 compared to other models. This is because Model 4 achieved the best balance between TPR and FPR in classifying the normal arteries. In addition, Model 2 which represents the second-order features showed the highest AUC value of 0.72 in classifying the non-calcified plaque as compared to other models. The poorest performance was reported for Model 3 which represents the shape-order features with the lowest AUC values of 0.50, 0.68, 0.58, and 0.62 in classifying the normal, calcified, non-calcified, and mixed plaques, respectively.

## 4. Discussion

Overall, there were three main findings based on the results as mentioned above. Firstly, our study highlighted that the first order features contributed the most in classifying the calcified and mixed types of atherosclerotic plaques, as well as differentiating the normal coronary artery from the abnormal ones. To our knowledge, no data describe the superiority of first order parameters in this situation. Moreover, this is opposed to the study by Gillies et al. in 2016, who emphasized that different lesions may have similar first order parameter values because the first order features describe the distribution of individual Hounsfield (HU) values without considering the spatial relationship [48]. It is believed that most probably the individual voxel values for the calcification composition in both calcified and mixed plaque are sufficient to provide statistical information on the distribution of HU values to differentiate between them even though without concern for the spatial relationship. For example, the calcified plaque is much easier to be differentiated due to its higher attenuation which is represented by its higher HU values in a voxel [49].

Secondly, our study emphasized that the second order features were especially useful for the classification of the non-calcified atherosclerotic plaque. The features of GLCM, GLRLM, NGLDM, and GLZLM were used to derive texture features or better known as second order features in our study. For instance, GLCM considers the arrangements of pairs of voxels to calculate the texture indices, and it represents the frequency of adjacent occurrences of a given value voxel pair [50]. GLRLM describes the size of homogeneous runs for each grey level while GLZLM provides the information on the size of homogeneous zones for each grey level in three dimensions. Moreover, NGLDM describes the difference of grey level between one voxel and its 26 neighbours in three dimensions. Therefore, these second order features describe the statistical relationships between the adjacent voxels, thereby can demonstrate the intra-lesion heterogeneity [21]. In terms of attenuation pattern, the non-calcified plaques can be further classified into either homogenous plaques that have a region with subtle HU difference or heterogeneous plaques that have at least two regions with significant HU difference [51]. For instance, the plaque with napkin-ring sign (NRS), also known as the heterogeneous plaque, has low-density voxels in the central area surrounded by higher values of voxels at the peripheral.

However, in this study, we just consider them under the category of non-calcified plaque. As a result of this kind of heterogeneous morphology as characterized by NRS plaque, features incorporating the spatial distribution of voxels have a better diagnostic performance specifically in classifying the non-calcified plaque. Our results are in line with the research associated with the identification of NRS plaque that has shown better predictive ability of using second order features (GLRLM, GLCM, and geometry-based parameters) instead of the first order parameters. Williams et al. (2019) have proved that the low attenuation non-calcified atherosclerotic plaque is more powerful in predicting myocardial infarction events due to its higher vulnerability of rupture [8]. Hence, our finding showed the possibility of using second order features in the future to build a classification model that can differentiate the non-calcified plaque which was found to be an important predictor for major cardiac events. Although Model 2 consisting of second order features achieved the best performance in classifying the non-calcified plaques compared to other features, its diagnostic performance in terms of precision, recall, and F1 score was considered suboptimal only and not in the best range. This was most probably associated with the subtle HU differences from the surrounding tissues that made the detection of the non-calcified plaques especially the homogeneous type became more challenging [52].

Thirdly, we found out that the shape order features did not contribute to the classification of any types of atherosclerotic plaques. Shape-based features represent the shape of the traced region of interest (ROI) and its geometric properties in terms of volume, sphericity, surface, and compacity [53]. Atherosclerotic plaques usually have complex irregular geometrical shapes [54]. Therefore, it may cause the shape order features most likely to become unrealistic to be used as one of the predictors in classifying the atherosclerotic plaques. Moreover, the composition and pattern of the atherosclerotic plaques that can characterize the type of plaques are much easier to be conveyed via the voxel intensities differences instead of the shape-based difference.

To the best of our knowledge, this is the first study to use TPOT as an AutoML approach to find the most appropriate pipeline for multi-class classification of atherosclerotic plaques of CCTA images. This was different from a prior study conducted by Kolossváry et al. in 2017 that employed the CCTA radiomics to binary classify the napkin ring sign vs. the non-napkin ring sign [51]. Furthermore, the difference was observed from the subsequent study by Kolossváry in 2019 that constructed a sum of eight radiomic-based ML models using different algorithms and finally selected the least angle regression model among the other 7 models due to its best result on the training set [55]. This model was then applied to the validation set for performance evaluation and comparison with conventional and histogram based CCTA analysis in differentiating between early and advanced atherosclerotic lesions. Therefore, there were two main contributions from our study. Firstly, radiomic features (first order, second order, and shape order features) extracted from the segmented coronary artery region were able to provide useful quantitative information for multi-class classification of the atherosclerotic plaques. Second, the process of classifying atherosclerotic plaques was totally automated using the TPOT method, which has been suggested as the best pipeline for model creation and subsequent clinical application.

Despite this, our research had a few drawbacks. To begin, CCTA imaging data were gathered from a single institution by following that institution’s imaging methodology. Several investigations have shown that CT scanner variability, reconstruction settings, and segmentation approaches all affect CT attenuation values, which in turn alter radiomic characteristics [56,57,58,59]. Secondly, the dataset was slightly imbalanced among each class of plaques. Therefore, ensuring a balanced dataset is strongly considered for study design in future CCTA image-based radiomics studies. In addition, sharing of imaging data across multiple centres with variety of classification model generated by other AutoML methods is recommended for simulation purposes. Other than that, with the latest trending of deep learning techniques [60] in this radiomics study, this research also promising for a better result in the next future.

## 5. Conclusions

Overall, our research showed that TPOT can suggest and adjust the best pipeline for each dataset when developing a machine learning model that can classify atherosclerotic plaques. Furthermore, we found out that the first order radiomic features contributed the most in differentiating the normal coronary arteries from arteries with atherosclerotic plaque, as well as in classifying the calcified and mixed plaques. Second order features were shown as a potential predictor in diagnosing the non-calcified plaques which have a high vulnerability to rupture. Besides that, we also showed that shape order features did not contribute to the classification of atherosclerotic plaques. Therefore, it is obvious that the characterization of atherosclerotic plaque must go beyond visual assessing the plaque and CCTA radiomics provides numerous quantitative information that enhances our current diagnostic and classification capabilities.

## Figures and Tables

**Table 1:** Demographic data of the study.

Characteristics	Total (*n* = 202)
**Patients’ Demographic Characteristics**	
Gender	
Male, *n* (%)	138 (68.3%)
Female, *n* (%)	64 (31.7%)
Ethnicity	
Malay, *n* (%)	104 (51.5%)
Chinese, *n* (%)	46 (22.8%)
Indian, *n* (%)	51 (25.2%)
Others, *n* (%)	1 (0.5%)
Age (years ± SD)	58.84 ± 9.497
Body Mass Index (kg/m^2^ ± SD)	26.81 ± 3.746
**Patients’ Distribution based on CAD-RADS**	
CAD-RADS 0, *n* (%)	6 (3.0%)
CAD-RADS 1, *n* (%)	9 (4.5%)
CAD-RADS 2, *n* (%)	54 (26.7%)
CAD-RADS 3, *n* (%)	36 (17.8%)
CAD-RADS 4, *n* (%)	95 (47.0%)
CAD-RADS 5, *n* (%)	2 (1.0%)
**Image Acquisition Characteristics**	
Total DLP (mGy × cm ± SD)	322.00 ± 167.926
Heart rate (bpm ± SD)	70.20 ± 10.522
Contrast medium (mL ± SD)	59.57 ± 2.347

**Table 2:** First order features (*n* = 29), second order features (*n* = 31), and shape order features (*n* = 5) are extensive descriptions of radiomic features collected from each divided region.

First Order Features (*n* = 29)	Second Order Features (*n* = 31)	Shape Order Features (*n* = 5)
**Conventional:** CONVENTIONAL_min CONVENTIONAL_mean CONVENTIONAL_std CONVENTIONAL_max CONVENTIONAL_Q1 CONVENTIONAL_Q2 CONVENTIONAL_Q3 CONVENTIONAL_Skewness CONVENTIONAL_Kurtosis CONVENTIONAL_Excess_Kurtosis CONVENTIONAL_peak_Sphere_0.5mL CONVENTIONAL_peak_Sphere_1mL CONVENTIONAL_calcium_AgatstonScore **Discretized:** DISCRETIZED_min DISCRETIZED_mean DISCRETIZED_std DISCRETIZED_max DISCRETIZED_Q1 DISCRETIZED_Q2 DISCRETIZED_Q3 DISCRETIZED_Skewness DISCRETIZED_Kurtosis DISCRETIZED_ExcessKurtosis DISCRETIZED_peakSphere0.5 mL DISCRETIZED_peakSphere1 mL DISCRETIZED_HISTO_Entropy_log^10^ DISCRETIZED_HISTO_Entropy_log^2^ DISCRETIZED_HISTO_Energy DISCRETIZED_AUC_CSH	**Gray Level Co-Occurrence Matrix (GLCM):** GLCM_Homogeneity GLCM_Energy GLCM_Contrast GLCM_Correlation GLCM_Entropy_log^10^ GLCM_Entropy_log^2^ GLCM_Dissimilarity **Gray Level Run length Matrix (GLRLM):** GLRLM_ Short Run Emphasis (SRE) GLRLM_Long Run Emphasis (LRE) GLRLM_Low Gray Run Emphasis (LGRE) GLRLM_High Gray Run Emphasis (HGRE) GLRLM__ Short Run Low Gray level Emphasis (SRLGE) GLRLM_ Short Run High Gray level Emphasis (SRHGE) GLRLM_Long Run Low Gray Level Emphasis (LRLGE) GLRLM_ Long Run High Gray Level Emphasis (LRHGE) GLRLM_GLNU (Gray-Level Non-Uniformity) GLRLM_Run-Length Non-Uniformity (RLNU) GLRLM_Run Percentage (RP) **Neighborhood Grey-Level Differences Matrix (NGLDM):** NGLDM_Coarseness NGLDM_Contrast NGLDM_Busyness **Grey Level Zone Length Matrix (GLZLM):** GLZLM_Short Zone Emphasis (SZE) GLZLM_Long Zone Emphasis (LZE) GLZLM_Low Grey-level Zone Emphasis (LGZE) GLZLM_ High Grey-level Zone Emphasis (HGZE) GLZLM_Short Zone High Grey-level Emphasize (SZHGE) GLZLM_ Long Zone Low Grey-level Emphasize (LZLGE) GLZLM_ Long Zone High Grey-level Emphasize (LZHGE) GLZLM_Gray-Level Non-Uniformity (GLNU) GLZLM_Zone-Length Non-Uniformity (ZLNU) GLZLM_Zone Percentage (ZP)	**Shape Features:** SHAPE Volume(mL) SHAPE_Volume(vx) SHAPE_Sphericity SHAPE_Surface(mm^2^) SHAPE_Compacity

**Table 3:** The diagnostic performance of each ML model in classifying the atherosclerotic plaques.

Atherosclerotic Plaques (Output)	ML Model	Recall (Sensitivity)	Precision (PPV)	F1- Score	Inverse Recall (Specificity)	Inverse Precision (NPV)	Inverse F1- Score	Accuracy
	1	0.97	0.80	0.88 *	0.90	0.99	0.94 *	0.92 *
Normal	2	0.83	0.73	0.77	0.88	0.93	0.90	0.86
	3	0.24	0.33	0.28	0.79	0.70	0.74	0.62
	4	1.00	0.72	0.84	0.85	1.00	0.92	0.89
	1	0.71	0.86	0.78 *	0.95	0.87	0.91 *	0.87 *
Calcified	2	0.66	0.82	0.73	0.93	0.85	0.89	0.84
	3	0.63	0.59	0.61	0.71	0.74	0.72	0.68
	4	0.74	0.74	0.74	0.88	0.88	0.88	0.83
	1	0.53	0.67	0.59	0.94	0.90	0.92 *	0.87 *
Non-calcified	2	0.58	0.69	0.63 *	0.94	0.91	0.92 *	0.87 *
	3	0.16	0.75	0.26	0.98	0.78	0.87	0.78
	4	0.37	0.78	0.50	0.98	0.87	0.92 *	0.87 *
	1	0.79	0.72	0.76 *	0.84	0.89	0.86 *	0.82 *
Mixed	2	0.79	0.69	0.74	0.81	0.88	0.84	0.80
	3	0.69	0.45	0.55	0.49	0.73	0.59	0.57
	4	0.72	0.74	0.73	0.86	0.85	0.85	0.81

The highest result of F1 score, Inverse F1 score, and accuracy was marked as (*).

## Data Availability

Not applicable.
